# Investigating the Prevalence of Menopausal Symptoms and Medico-Social Dimensions of Menopause in Rural Puducherry, India: A Cross-Sectional Analytical Study

**DOI:** 10.7759/cureus.55841

**Published:** 2024-03-09

**Authors:** Nishanthini Natarajan, Partha Nandi, Narayan KA, Lokeshmaran S

**Affiliations:** 1 Department of Community Medicine, Aarupadai Veedu Medical College, Puducherry, IND; 2 Department of Community Medicine, Mahatma Gandhi Medical College and Research Institute, Puducherry, IND

**Keywords:** socio-cultural factors, social dimensions, post menopause, menopause symptoms, menopause

## Abstract

Introduction

Globally, the health of women has been of great concern for healthcare providers for the past many years. However, the concern is limited to maternal and adolescent health and the life course approach is lagging. Thus, a need was felt to study the changes after menopause and its influential factors by determining the prevalence of menopausal symptoms and medico-socio-cultural dimensions of menopause among perimenopausal and early postmenopausal women.

Methodology

A cross-sectional analytical study was conducted from February 2014 to April 2015 in a rural area of Puducherry among 148 women in perimenopausal and early postmenopausal stages by complete enumeration using a pre-tested semi-structured questionnaire. Prevalence was expressed as frequency with percentage and the chi-square test was used to find the association among the study variables.

Results

The prevalence of menopausal symptoms was in 143 (96.6%) women. Backache (62.3%) was found to have a higher prevalence. Physical symptoms (93.2%) were most prevalent. Of the women, 95% were affected by mild symptoms. Regarding medical and social dimensions of menopausal symptoms, socioeconomic status to vasomotor, age of menarche to physical, marital status, and abortion status to sexual symptoms were significant. A positive correlation was seen among the four menopausal symptom domains, except for vasomotor to sexual symptoms.

Conclusion

Increased prevalence of menopausal symptoms is seen among menopausal women who are unaware of seeking medical advice. Additional emphasis should be placed on implementing programmes that will critically help to sensitize and intensify the awareness of menopause among women in India.

## Introduction

Globally, the health of women has been of great concern for healthcare providers for the past many years [1]. Firstly, the concern regarding women’s health in India was focused mainly on maternal health and contraception; however, in recent years, adolescent health has also gained importance due to the initiation of the Adolescent Reproductive and Sexual Health (ARSH) programme. However, issues over women’s health beyond the reproductive age have remained neglected till now, hence the life course approach is lagging [2]. Menopause is the period in the life of women during which the capacity to reproduce ceases or, in other words, a woman enters into a non-reproductive phase from the reproductive phase. These physiological changes turn out to be pathological changes resulting in decreased bone mineral density (osteoporosis), vaginal dryness (dyspareunia), mood disorders, etc. [3]. Secondly, there is no standard data related to postmenopausal women in India, especially from rural areas, which is the place where most of the women of this age group are expected to reside and it is the place where there is a lack of services to these women [4]. As the current geriatric healthcare services are essentially towards the overall health problems of the elderly, no special direction has been headed for the women in the postreproductive stage. According to the current estimates (2012), life expectancy for women is 72.7 years as against 68.1 years for males [5]. With this increase in life expectancy, a woman spends about one-third or two and a half decades of her life after menopause. It makes postmenopausal life as significant as before the menopause [6]. Postmenopausal women are the most vulnerable group affected due to physiological changes. Researchers have found that the oestrogen level that decreases during menopause is the reason for menopausal symptoms, which affect the physical and psychological well-being of menopausal women, which they are unaware of [7]. Thus a need was felt to study the changes taking place in women after menopause and also the influential factors of menopause. This study aims to determine the prevalence of menopausal symptoms among perimenopausal and early postmenopausal women and also to assess the medico-social dimensions of menopause among them.

## Materials and methods

A cross-sectional analytical study was conducted in Seliamedu, a rural field practising area of a tertiary care hospital, for a period of one year and six months from February 2014 to August 2015 among perimenopausal and early postmenopausal women. The study was conducted after obtaining ethical clearance from the Institutional Human Ethics Committee, Mahatma Gandhi Medical College and Research Institute (PG/2014/08). The study included all women over 40 years of age who had lived in the selected rural area for more than a year and whose last menstrual cycle was regular and lasted no longer than five years. Women who had undergone hysterectomy, received hormonal therapy, were seriously ill, physically challenged, deaf & dumb, and women who were not in their house for three successive visits were excluded from the study. The Stages of Reproductive Aging Workshop (STRAW) staging system for reproductive ageing in women (2012) was used to classify the women [8]. Prior to the start of the study, a pilot study was done at the nearby village (Pinnachikuppam) with 30 subjects to assess the feasibility and reliability of the questionnaire. Cronbach’s alpha coefficient was 0.842.

Complete enumeration of perimenopausal and early postmenopausal women was done by house-to-house survey using a pre-structured and pre-tested semi-structured questionnaire, which was in the local Tamil language. Visits were done for one year. So at the end of the study, 148 women were interviewed by universal sampling.

The questionnaire consists of three parts. Part I includes socio-demographic data like age, religion, parity, marital status, occupational status, type of family, and socio-economic status. Part II consists of a questionnaire on menopausal symptoms. The menopausal symptom questionnaire consists of 25 individual items, which were then grouped under four domains (vasomotor, psychosocial, physical, and sexual) as per the menopause-specific quality of life questionnaire [9]. Symptoms were recorded on a five-point Likert scale used in the World Health Organization Quality of Life (WHOQOL) questionnaire [10], and the severity was assigned as follows: 0 - no symptoms; 1 - symptoms present but not bothersome (mild); 2 - symptoms present, bothersome, not affecting physical activity (moderate); 3 - symptoms present, extremely bothersome, not affecting physical activity (severe); 4 - symptoms present, extremely bothersome and affecting daily activity (very severe). For analysis of sexual symptoms, 25 widows whose husbands died before they attained menopause were excluded. For medical dimensions, abortion, menopausal symptoms, menstrual irregularities, and history of diabetes or hypertension were considered.

Data were entered in a Microsoft Excel sheet (Microsoft Corporation, Redmond, WA) and analysis was done in Epi Info version 6 (CDC, Atlanta, GA) and SPSS version 26 software (IBM Corp., Armonk, NY). Prevalence of menopausal symptoms was expressed as the frequency with percentage. The chi-square test was used to find the association among the study variables. Spearman's rank correlation analysis was done to find the association of the ordinal variable as it does not follow a normal distribution. P-value < 0.05 was considered as statistically significant.

## Results

This study was done at Seliamedu village, a rural field practising area of a tertiary care hospital, with an aim to determine the prevalence of menopausal symptoms among perimenopausal and early postmenopausal women. A total of 1390 houses were visited, which covers a population of about 5485, among whom 2919 (53.2%) were females. Among them, 1900 (65%) women were below 40 years of age and 1019 (35%) were above 40 years of age. A total of 148 women (14.5%) who were fulfilling our study criteria were interviewed.

Among the interviewed women, 28 were in the perimenopausal stage and 120 were in the early postmenopausal stage. The mean age of attaining menopause was 47.8 ± 3.7 years. Among the 148 women, 20 (13.5%) were in the age group of 40 to 45 years, 60 (40.5%) were in the age group of 46 to 50 years, 57 (38.5%) were in the age group of 51 to 55 years, and only 11 (7.5%) were above 55 years of age. About 84 (56.8%) women were illiterate, 33 (22.8%) had primary level education, and 31 (20.4%) had secondary level education. Regarding the occupational status of the women in the study, 86 (58.1%) were working while the rest were housewives. The majority (71.6%) of the women were living in a nuclear family and the remaining 28.4% were living in a joint family. Of the women, 72 (49%) attained menopause at the age of 46 to 50 years and eight (5%) women had premature menopause before 40 years of age (Table 1).

**Table 1:** Reproductive characteristics of the study population (n = 148) * Modified BG Prasad scale.

Characteristics		Frequency	Percentage
Age of menarche	Early	32	21.6
	Ideal	65	43.9
	Late	51	34.5
Marital status	Unmarried	1	0.7
	Married	56	79.1
	Widow	29	20.2
Socio-economic status*	Upper	2	1.4
	Upper middle	13	8.7
	Middle	25	16.9
	Lower middle	46	31.1
	Lower	62	41.9
Parity	Nulliparous	8	5.4
	1-3 child	122	82.4
	>3	18	12.2
Abortion	No	107	72.3
	Yes	41	27.7
Menopausal status	Perimenopause	28	18.9
	Postmenopause	120	81.1
Menstrual cycle	Regular	71	48
	Irregular	77	52
Age of menopause	<41 years	8	5.4
	41-45 years	38	25.7
	46-50 years	72	48.6
	>50 years	30	20.3

Table 2 shows the frequency distribution of menopausal symptoms among menopausal women. The overall prevalence of menopausal symptoms was 143 (96.6%). Among the menopausal symptoms, backache was found to have a higher prevalence, which is about 93 (62.3%). Dysuria was found to have the lowest prevalence in 13 (8.8%) women. Among the psychosocial symptoms, forgetfulness was found in 81 (54.6%) and anxiousness in 37 (25%) women. Increased level of vasomotor symptoms was seen in waking up at night in 60 (40.5%), followed by night sweats in 49 (33.1%), and lastly hot flushes in 45 (30.4%). For sexual symptoms, those widows (3%) whose husbands died after attaining menopause were included in this analysis. Among the sexual symptoms, reduced sexual desire was found in 56 (37.8%), followed by avoiding intimacy in 52 (35.8%), and lastly, dyspareunia was reported by 35 (23.6%) women. About 138 (93.2%) women had reported physical symptoms, followed by psychosocial symptoms, which accounts for about 119 (80.4%). Third is the vasomotor symptom, reported by 84 (56.8%) women and lastly sexual symptoms, reported by 62 (50.4%) women.

**Table 2:** Prevalence of menopausal symptoms among the subjects (n = 148) * A total of 20.3% of widows were excluded. Among those widows, 3% of women’s husbands died after they attained menopause.

Menopausal symptoms	Domains	Present, n (%)	Absent, n (%)
Hot flushes	Vasomotor	45 (30.4)	103 (69.6)
Night sweats		49 (33.1)	99 (66.9)
Waking up at night		60 (40.5)	88 (59.5)
Depression	Psychosocial	69 (46.6)	79 (53.4)
Forgetfulness		81 (54.7)	67 (45.3)
Anxiousness		37 (25)	111 (75)
Lack of concentration		60 (40.5)	88 (59.5)
Irritability		69 (46.6)	79 (53.4)
Fatigability	Physical	84 (56.8)	64 (43.2)
Body ache		89 (60.1)	59 (39.9)
Joint pain		90 (60.8)	58 (39.2)
Backache		93 (62.8)	55 (37.2)
Sleeplessness		70 (47.3)	78 (52.7)
Headache		60 (40.5)	88 (59.5)
Urinary frequency		55 (37.2)	93 (62.8)
Urinary urgency		58 (39.2)	90 (60.8)
Burning micturition		26 (17.6)	122 (82.4)
Urinary incontinence		39 (26.4)	109 (73.6)
Dysuria		13 (8.8)	135 (91.2)
Dizzy spells		51 (34.5)	97 (65.5)
Feeling bloated		51 (34.5)	97 (65.5)
Gas pain		30 (20.3)	118 (79.7)
Reduced sexual desire	Sexual^*^ (N = 123)	56 (45.5)	67 (54.5)
Dyspareunia		35 (28.5)	88 (71.5)
Avoiding intimacy		52 (42.3)	71 (57.7)

**Figure 1:**
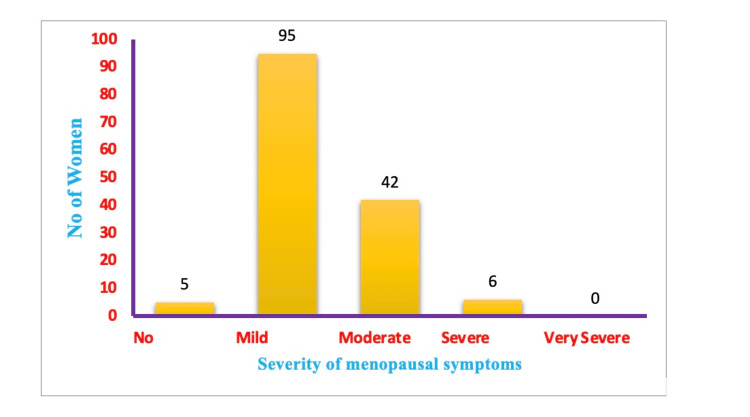
Prevalence of menopausal symptoms in accordance with the severity (n = 148)

Figure 1 shows the prevalence of menopausal symptoms according to their severity. About five (3.4%) menopausal women have no menopausal symptoms and there were no women with very severe menopausal symptoms. A total of 95 (64.2%) women have experienced mild form of menopausal symptoms. Among the studied women, 28.4% had moderate form of symptoms.

Table 3 shows the association between the severity of symptoms and the medical social dimensions. A significant association was seen between the socio-economic status and the severity of the vasomotor symptoms, the age of menarche and physical symptoms, and sexual symptoms with marital status and abortion. As the age of menarche increases, the severity of the physical symptoms also increases. Other medical and social dimensions were not significantly associated with the severity of the symptoms.

**Table 3:** Association of the severity of symptoms with medical and social dimensions (n = 148)

	No symptoms (n = 64)	Mild (n = 41)	Moderate (n = 26)	Severe (n = 14)	Very severe (n = 3)	Total	Chi-square value	P-value
Socio-economic status - vasomotor								
Class I	1 (50)	0 (0)	0 (0)	0 (0)	1 (50)	2	40.94	0.001
Class II	2 (15.4)	7 (53.8)	1 (7.7)	2 (15.4)	1 (7.7)	13		
Class III	12 (48)	9 (36)	1 (4)	3 (12)	0 (0)	25		
Class IV	29 (46.8)	13 (21)	14 (22.6)	6 (9.7)	0 (0)	62		
Class V	20 (43.5)	12 (26.1)	10 (21.7)	3 (6.5)	1 (2.2)	46		
Age of menarche - physical								
Early	2 (6.3)	22 (68.8)	8 (25)	0 (0)	0 (0)	32	24.7	0
Ideal	8 (12.3)	33 (50.8)	24 (36.9)	0 (0)	0 (0)	65		
Late	0 (0)	29 (56.9)	14 (27.5)	8 (15.)	0 (0)	51		
Marital status - sexual								
Unmarried	1 (10.6)	0 (0)	0 (0)	0 (0)	0 (0)	1	24.1	0.002
Married	56 (47.9)	29 (24.8)	23 (19.7)	7 (6)	2 (1.7)	117		
Widow	29 (96.7)	1 (3.3)	0 (0)	0 (0)	0 (0)	30		
Abortion - sexual								
No	49 (63.6)	22 (20.6)	12 (11.2)	5 (4.7)	0 (0)	88	11.858	0.015
Yes	12( 43.9)	8 (19.5)	11 (26.8)	2 (4.9)	2 (4.9)	41		

Table 4 shows the correlation matrix of the menopausal symptoms. In this study, a significant positive correlation is seen among all three domains of menopausal symptoms (vasomotor, psychosocial, and physical) (p < 0.01). Sexual symptoms were positively correlated (p < 0.05) with physical and psychosocial symptoms, except for vasomotor symptoms.

**Table 4:** Correlation analysis of four domains of menopausal symptoms ** Correlation is significant at the 0.01 level (two-tailed). * Correlation is significant at the 0.05 level (two-tailed).

		Vasomotor	Psychosocial	Physical	Sexual
Vasomotor	Correlation coefficient	1			
	Sig. (2-tailed)	.			
Psychosocial	Correlation coefficient	0.371**	1		
	Sig. (2-tailed)	<0.001	.		
Physical	Correlation coefficient	0.522**	0.620**	1	
	Sig. (2-tailed)	<0.001	<0.001	.	
Sexual	Correlation coefficient	0.166	0.185*	0.192*	1
	Sig. (2-tailed)	0.067	0.04	0.033	

## Discussion

In the current study, the mean age of attaining menopause was 47.8 ± 3.7 years, which was closer to the age of menopause given by the Indian Menopause Society (47.9 years) [11]. In the present study, married women were 79%, widows were 20%, and a single woman was unmarried. A similar distribution was seen in a study conducted in Vadodara district with 147 women, of which 80.3% were married and 18.4% were unmarried [4].

Tom et al. conducted a cross-sectional study in the US among 2397 women and showed that the mean age of menopause was 49 years [12]. Dratva et al. published data from a European cohort study showing that the mean age of menopause was 54 years [13]. Thus a wide range of variation in the mean menopausal age was noticed, which seems to be due to the diversity in nation, race, ethnicity, and environmental factors.

In the present study, about 96.6% of the women were experiencing menopausal symptoms, except for five women who did not have menopausal symptoms. Singh et al., in their study done in New Delhi, showed that above 90% of women experienced one or the other symptom of which they were aware but were not seeking health care [14]. In the present study, muscles and joint pain (60% to 63%) were the common menopausal symptoms experienced by the study subjects, which were similar to the symptoms experienced by Asian women, unlike Western women. A similar finding was seen in a cross-sectional study conducted by Amrita et al. and Poomalar et al., where muscles and joint pain predominated but the percentage was less than 60% [15,16]. Sagdeo et al., in their study among rural women in Nagpur, showed muscle and joint pain had a higher prevalence [17]. Similar findings were also observed in a study conducted in Pondicherry by Poomalar et al. but the percentage was less than 60% [16].

Among all four domains, physical symptoms (93.2%) were found to be higher in prevalence compared to those of other symptoms. In general, vasomotor symptoms are more prevalent among Western women but Asian women experience physical symptoms more for multiple reasons, as shown in various studies. Loutfy et al. found that 90.7% of women were complaining of hot flushes, which were due to the hot climate in their study area [18]. The increased intake of phytoestrogens by Asian women, which maintains their oestrogen level, helps to cope with menopausal syndrome. Nisar et al. had a different finding of Pakistani women experiencing physical symptoms (99%) at a higher rate than the other symptoms in the prevalence of psychological symptoms (96%), vasomotor symptoms (71%), and sexual symptoms (66%) [19]. Nayak et al., in their study conducted in a coastal area of Karnataka among 209 women, found significant differences in all the symptoms, except for sexual symptoms among the perimenopausal and postmenopausal women [20]. Punyahotra et al. also showed that a significant association was seen in muscle and joint pain with postmenopausal women [21].

The limitations of the study include recall bias where women were asked to recall their symptoms in the past, which would have led them to give false information in case they would not have recalled appropriately, and being a cross-sectional study, the cause-and-effect relationship could not be identified.

Thus, menopause and its symptoms had variability among nation, race, and individual perceptions. It was influenced by multiple factors like age, socioeconomic status, psychological factors, socio-cultural factors, peer group effect, diet, and lifestyle. It is advised to form peer groups to discuss and share their issues on menopause and menopausal clinics to dispense advice on how to navigate this period of life with lifestyle changes, professional help, and guidance. Reproductive and child health programme can be further extended towards menopausal problems and help for the betterment of menopausal women.

## Conclusions

In the current study, a higher prevalence of menopausal symptoms was found. Muscles and joint pain were the most common symptoms and dysuria was the least common symptom experienced by the participants. The present study showed that there is a need to address the burden of menopausal symptoms as the prevalence is very high by creating awareness about menopausal symptoms and conducting health educational programmes on menopause, counselling, and other forms of health services. In conclusion, this study contributes valuable insights into the prevalence and dimensions of menopausal symptoms among perimenopausal and early postmenopausal women in a rural setting. The findings underscore the importance of adopting a comprehensive approach to women's health that extends beyond reproductive years. Addressing menopausal symptoms requires not only medical interventions but also consideration of socioeconomic, cultural, and marital factors. This research provides a foundation for future studies and interventions to improve women's holistic well-being during and after menopause.
